# Linseed Oil-Based Oleogel Vehicles for Hydrophobic Drug Delivery—Physicochemical and Applicative Properties

**DOI:** 10.3390/pharmaceutics16050600

**Published:** 2024-04-29

**Authors:** Sonia Kudłacik-Kramarczyk, Anna Drabczyk, Alicja Przybyłowicz, Marcel Krzan

**Affiliations:** 1 Jerzy Haber Institute of Catalysis and Surface Chemistry, Polish Academy of Sciences, 8 Niezapominajek St., 30-239 Krakow, Poland; alicja.przybylowicz@student.pk.edu.pl (A.P.); marcel.krzan@ikifp.edu.pl (M.K.); 2 CBRTP SA—Research and Development Center of Technology for Industry, Ludwika Waryńskiego 3A St., 00-645 Warsaw, Poland; 3 Faculty of Mechanical Engineering, Cracow University of Technology, 37 Jana Pawła II Av., 31-864 Krakow, Poland

**Keywords:** oleogels, rheology, beeswax, Tween^®^20, Tween^®^80, emulsifier, surface wetting

## Abstract

In this study, a methodology for synthesizing oleogels based on linseed oil and emulsifiers, such as beeswax and Tween 20 and Tween 80, was developed. Linseed oil served as the main oil phase, while beeswax acted as a gelling and emulsifying agent. Tween compounds are non-ionic surfactants composed of hydrophobic and hydrophilic parts, allowing for the formation of a stable system with promising properties. Surface wetting analysis of the obtained oleogels, FT-IR spectroscopy, and determination of relative and absolute humidity over time, as well as optical microscope analysis and rheological analysis of the obtained oleogels, were conducted as part of the research. The results indicate that increasing the amount of Tween 20 decreases the hydrophilicity of the oleogel, while Tween 80 exhibits the opposite effect. Surface energy analysis suggests that a higher content of Tween 20 may lead to a reduction in the surface energy of the oleogels, which may indicate greater material stability. Changes in relative humidity and FT-IR spectral analysis confirm the influence of emulsifiers on the presence of characteristic functional groups in the structure of the oleogels. Additionally, microscopic analysis suggests that an emulsifier with a longer hydrophobic tail leads to a denser material structure.

## 1. Introduction

Oleogels are materials with a semi-solid consistency, containing a significant amount of liquid oil enclosed within a network of structural molecules. In such a system, oil molecules act as the dispersed oil phase [1,2]. A characteristic feature of oleogels is their ability to maintain stability and homogeneity, facilitating their effective use as carriers for active substances across various fields including pharmacy [3], cosmetology [4], and the food industry [5,6,7].

In cosmetology, oleogels are widely used as components in many skincare and haircare products. Their ability to form a protective layer makes them excellent at moisturizing the skin while protecting it from water loss and negative external factors [8,9]. In pharmacy, oleogels primarily serve as carriers for active substances in the form of medications. Due to their hydrophobic nature, they can transform and release both lipophilic and hydrophilic substances, making them versatile in therapy. In addition, their flexible consistency enables the controlled release of active substances, which is crucial for extended-release drugs [10,11,12].

Within the scope of this study, oleogels were developed based on beeswax, linseed oil, and emulsifiers, namely Tween 20 and Tween 80. Linseed oil is a colorless or slightly yellow liquid serving as a rich source of fatty acids, primarily alpha-linolenic acid (ALA), linoleic acid (LA), and oleic acid (OA), constituting approximately 90% of its composition. In addition, it contains phytosterols, tocopherols (vitamin E), and other biologically active substances [13,14,15]. Beeswax, on the other hand, is primarily a mixture of fatty acid esters (e.g., palmitic acid, myristic acid) and polyhydroxyl alcohols (e.g., triterpenes, aliphatic alcohols), alongside other components such as carboxylic acids and vitamins. It finds use as a gelling agent or emulsifying agent [16,17,18].

Based on a literature review, oleogels comprising linseed oil, beeswax, and either Tween 20 or Tween 80 have not been previously explored. In the context of oleogel formulation, linseed oil serves as the primary oil phase, while beeswax acts as a gelling agent and emulsifier. Meanwhile, Tween surfactants (polysorbates) are non-ionic surfactants featuring a hydrophobic sorbitan moiety and hydrophilic polyoxyethylene chains [19,20,21]. As a result, the combination of linseed oil, beeswax, and either Tween 20 or Tween 80 can contribute to the development of a stable oleogel with promising applications.

In the present study, oleogels based on linseed oil and beeswax mixed in a weight ratio of 1:5 were obtained, with two series of syntheses—one employing Tween 20 and the other employing Tween 80. Subsequently, rheological analysis, wetting characterization towards both polar and non-polar liquids, and sorption properties examination were carried out for the obtained materials. Additionally, the chemical structure of the oleogels was characterized using FT-IR spectroscopy, while their morphology was analyzed using an optical microscope. The analyses primarily focused on assessing the impact of the type and amount of Tween utilized on the physicochemical properties of the oleogels.

## 2. Materials and Methods

### 2.1. Materials

Main component of oleogel was linseed oil (*Linum usitatissimum* seed oil). Linseed oil is produced by pressing the seeds of the common flaxseed (Linum usitatissimum) wherein these seeds are the main source of flaxseed oil. This oil contains high levels of essential fatty acids (both omega-3 and omega-6) and linolenic acid (approx. 57%). Additionally, it also contains oleic acid, linoleic acid, palmitic acid, and stearic acid [22]. Linseed oil LenVitol was purchased from Oleofarm LLC (Wrocław, Poland). Refined beeswax (Cera alba) was bought in Sigma Aldrich (Saint Louis, MO, USA). Tween^®^ 20 (Polysorbate, reagent grade, d = 1.100 g/mL) and Tween^®^ 20 (Polysorbate, proteomics grade, d = 1.062 g/mL) were purchased from VWR International Sp. z o.o (Gdańsk, Poland). All reagents were used as received without further purification.

### 2.2. Preparation of Leenseed Oil-Based Oleogel

The first step in obtaining oleogel was to introduce appropriate amounts of linseed oil and beeswax into the beaker wherein the whole was next placed on a magnetic stirrer, heated to 120 °C, and subjected to intense stirring (1200 rpm) to fully dissolve the beeswax and obtain a homogeneous mixture. Next, the mixture obtained was cooled to 60 °C and supplemented with an adequate amount of Tween^®^ 20 or Tween^®^ 80. The whole was subsequently reheated to 120 °C and homogenized (4000 rpm, 15 min) using a Unidrive X1000 CAT homogenizer (CAT, M. Zipperer GmbH, Ballrechten-Dottingen, Germany) to obtain a uniform mixture.

**Figure 1:**
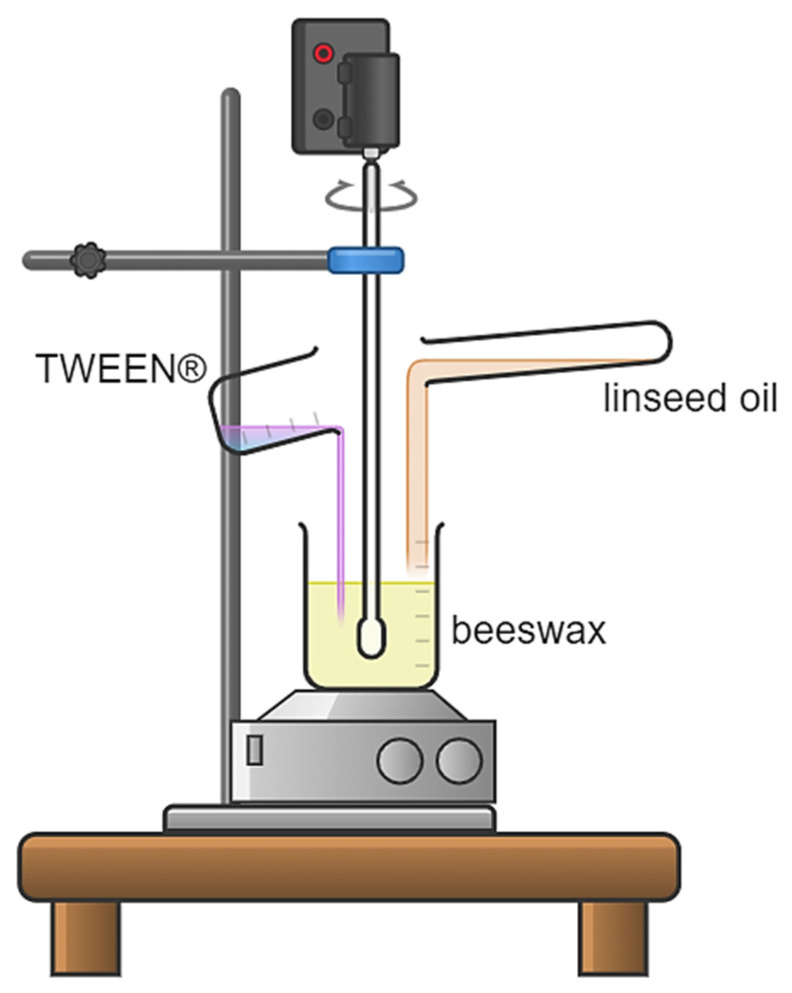
Scheme of obtaining semi-solid emulsions based on beeswax and linseed oil.

The scheme of the process is presented below in Figure 1.

**Figure 2:**
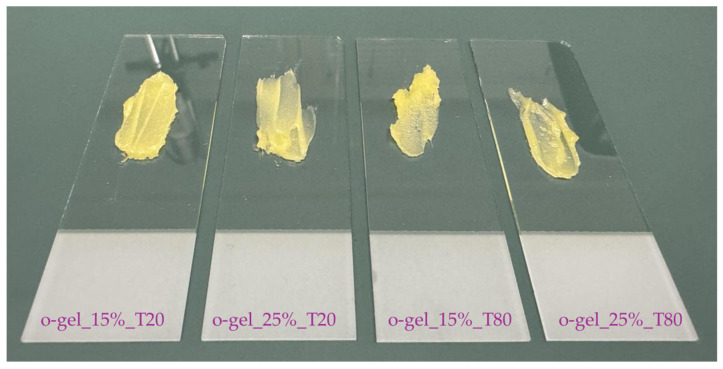
Photographs illustrating the obtained oleogels.

The compositions of all the prepared oleogels are compiled below in Table 1. In turn, in Figure 2, images of the obtained oleogels are presented.

Finally, the systems obtained were poured into small vessels and subjected to further experiments aimed at verifying their physicochemical properties.

### 2.3. Studies on the Wettability of the Developed Systems

An essential part of the performed experiments was to determine the wetting properties of the obtained oleogels. For this purpose, the Drop Shape Analyzer Kruss DSA100M optical contact angle measuring instrument (A. KRÜSS Optronic GmbH, Hamburg, Germany) was employed. Sterile syringe needles (NE 44, Kruss Gmbh, Hamburg, Germany) were applied for each new measurement. The Kruss DSA100M is equipped with an optical microscope and a digital camera (200 fps) which takes photographs of the analyzed samples and, importantly, applies a digital image processing algorithm to calculate the contact angles using Laplace–Young approximations or tangents. The applied measuring instrument is known for accurate measurement results, simple operation, and high precision. During the experiments, the temperature of the test chamber is controlled via a thermostatic water bath, wherein both humidity (43%) and temperature conditions (22.0 ± 0.3 °C) are maintained at a constant level.

Before the analysis, samples of the prepared oleogels were spread on the test platform inside the environmental cell with a fixed syringe. Next, the needle position was adjusted. Each oleogel sample was spilling over the test platform forming a layer, thus it was necessary to wait 10 s until the position of the sample was stabilized. Then, the contact angles were recorded on the video and measured (after determining the baseline). After the liquid applied to the sample had stabilized, the device displayed the calculated contact angle values. The same procedure was employed in the case of all investigated oleogel layers wherein for each tested material, three measurements were conducted. Importantly, the contact angles were determined for two liquids, i.e., polar (pure double distilled water, Millipore Q, 18.60 mW/cm, δ = 72.30 mN/m) and nonpolar (diiodomethane, δ = 50.80 mN/m). Before the experiments, surface tension measurements aimed at verifying the water purity were performed. The equilibrium surface tension of pure double distilled water was determined using the Drop Shape Analyser DSA100 (A. KRÜSS Optronic GmbH, Hamburg, Germany) in line to the pendant drop method carried out using sterile syringe needles (A. KRÜSS Optronic GmbH, Hamburg, Germany).

Additionally, the performed experiments allowed us to determine the surface free energy of the investigated oleogel samples. Here, the Owens–Wendt method was employed, which is based on the assumptions that the interactions occurring between molecules of two substances at their interface are equivalent to the geometric mean of the intermolecular interactions within each substance individually. Such an applied methodology makes it possible to calculate both the dispersive and polar component of the surface free energy using the following Equations (1) and (2):

(1) $${\left(\gamma_{S}^{D}\right)}^{0.5}=\frac{\gamma_{d}\;\left(cos\theta_{d}+1\right)-\sqrt{\frac{\gamma_{d}^{p}}{\gamma_{w}^{p}}\gamma_{w}(cos\theta_{w}+1)}}{2\;\sqrt{\gamma_{d}^{D}-\sqrt{\gamma_{d}^{P}}\frac{\gamma_{w}^{D}}{\gamma_{w}^{P}}}}$$

(2) $${\left(\gamma_{S}^{P}\right)}^{0.5}=\frac{\gamma_{dw}\;\left(cos\theta_{w}+1\right)-2\sqrt{\gamma_{S}^{D}\gamma_{w}^{D}}}{2\;\sqrt{\gamma_{w}^{P}}}$$

A detailed description of this approach has been presented in [23].

### 2.4. Sorption Properties of Oleogels Based on Relative Humidity (RH) Changes

**Figure 3:**
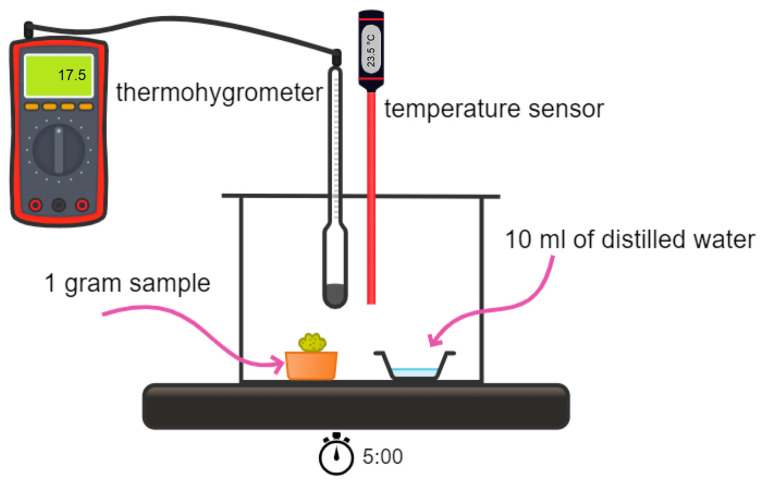
Scheme of humidity examination of received materials.

The sorption capacity of the developed materials was characterized by verifying the changes in ambient relative humidity in the presence of the tested oleogels and the corresponding liquid, i.e., pure double deionized water. For this purpose, 1.0 g of oleogel sample was weighed, placed on a Petri dish, and transferred to a Duran glass bottle. Next, 10 mL of pure double deionized distilled water was poured into the other Petri dish and placed near the Petri dish with the tested oleogel sample. Finally, the glass bottle was tightly closed, wherein a humidity sensor was placed so that it did not come into direct contact with either Petri dish. Such a measurement was performed for 30 min for each sample wherein changes in both humidity and temperature were noted every 5 min. Each experiment was initiated at a starting temperature of 23.5 °C. At the end of each experiment, the Duran glass bottle was cleaned, dried in a laboratory dryer at 30 °C, then left until it reached 23.5 °C. The scheme of the experiment is presented below in Figure 3.

Measurements were performed using a thermo/hydrometer Elmetron PWT-401 (Elmetron GP, Zabrze, Poland). The device allows for simultaneous measurements of relative humidity and temperature (relative humidity range 0–100%; resolution 0.1% RH).

Based on the determined values of relative humidity, the absolute humidity (AH) can be calculated using Equation (3):

(3) $$AH=\left[\left(\frac{1320.65}{T+273.15}\right)\times 10\;\left(\frac{7.4475\;T}{T+233.71}\right)\right]\times RH$$

where

- AH is absolute humidity, kg/g;
- RH is relative humidity, %.

### 2.5. Characterization of Oleogels via FT-IR ATR Spectroscopy

In order to determine the functional groups within the structure of the obtained oleogel samples, Fourier transform infrared (FT-IR) spectroscopy was employed. Analysis was performed using a Thermo Scientific Nicolett iS10 (Thermo Fisher Scientific Inc., Waltham, MA, USA) spectrometer equipped with an attenuated total reflection (ATR) attachment containing a germanium crystal. The measurement range was 4000–1500 cm^−1^ (32 scans at 0.4 cm^−1^ resolution). Measurements were performed at ambient temperature.

### 2.6. Imaging of Oleogels by Means of Optical Microscopy

Oleogels were additionally imaged using a BRESSER Researcher Trino 40-1000x (Bresser, Rhede, Germany) optical microscope. The microscope is equipped with a Moticam Digital Camera 3.0 MP (Motic, San Antonio, TX, USA) wherein the images were recorded using Motic Image Plus Version 2.0 ML software (Motic, San Antonio, TX, USA). Imaging was performed at ambient temperature.

### 2.7. Rheological Properties of Oleogels

The rheological properties of the obtained materials were tested using the Malvern Bohlin Gemini II (Malvern Panalytical Ltd., Cambridge, UK) rheometer equipped with the sample temperature monitoring using a Peltier system and the software Bohlin R6.51.0.3 (Malvern Panalytical Ltd., Cambridge, UK) dedicated to this equipment. The measurement temperature was 25 ± 1 °C. The amount of oleogel used was about 1 g, and the gap between the cone and plate probe (diameter = 40 mm, angle = 4°) was set to 1000 µm. The complex shear modulus was measured as a function of increasing applied strain amplitude at a fixed frequency equal to 1 Hz. The complex shear modulus, denoted G* = G′ + iG″, is obtained from the amplitudes and phases of the first harmonic strain signals. Close to the yield strain, the rheological response is nonlinear, so that the full spectrum of strain or stress harmonics must be considered for a complete study of the behavior. The complex shear modulus was measured as a function of the controlled strain. At least three measurements of rheological properties were performed for each developed hydrogel. In the publication, we present the results of the measurement whose curves were closest to the mathematically determined average of all measurement results. A frequency sweep test (from 0 to 10 Hz) was used to determine the elastic modulus (G′) and loss modulus (G″) of the samples in the linear viscoelastic region (LVR).

## 3. Results

### 3.1. Results of Wettability Analysis

Images of water drop contact with the tested oleogel layers are presented in Table 2, whereas Table 3 shows the values of the measured contact angles of oleogels, as well as the values of the calculated surface free energies (including dispersive energy, polar energy and total free energy).

The results of the wettability measurements indicate that the amount of Tween used during the synthesis of oleogels affects their wettability. In the case of samples containing Tween 20, the greater the amount of this emulsifier, the greater the wetting angle, and therefore the lower the wettability. On the other hand, an inverse relationship was reported for samples obtained with Tween 80—the sample o-gel_25%_T80 is characterized by greater hydrophilicity (smaller wetting angle) than the sample o-gel_15%_T80.

In turn, analysis of the surface free energies of oleogels suggests that a higher Tween content may have the effect of reducing or increasing (depending on the type of Tween) the surface stability of oleogels. In addition, the type of Tween applied may also influence the surface stability. Nonetheless, this effect may be limited compared to differences in the Tween content in the samples.

### 3.2. Sorption Properties of Oleogels Verified via the Ambient Humidity Changes

**Figure 4:**
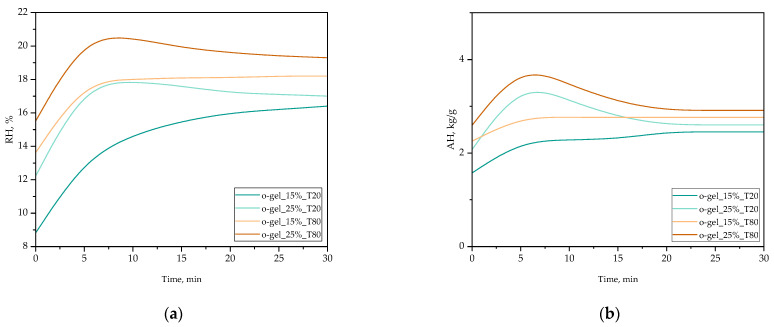
(**a**) Changes in relative humidity (RH) in the test material over 30 min. (**b**) Changes in absolute humidity (AH) in the test material over 30 min.

Changes in the relative humidity over 30 min of performed experiments are presented in Figure 4a. The absolute humidities calculated in line with adequate equations and using the values of RH are displayed in Figure 4b.

Based on the results of performed investigations, it can be concluded that both the type of Tween used during the synthesis of oleogels and its amount affect the values of relative humidity reported for the tested samples, and thus their sorption properties. The initial relative humidities of the o-gel_25%_T80 and o-gel_15%_T80 samples were higher than the values of this parameter reported for the o-gel_25%_T20 and o-gel_15%_T20 samples. Importantly, the significantly highest initial relative humidity value—approximately 20.5%—was determined for the o-gel_25%_T80 sample. Furthermore, comparing the relative humidities measured for samples obtained using the same Tween, it can be observed that the higher the content of this emulsifying agent in the investigated oleogel, the higher the relative humidity of the tested sample, and thus the lower the sorption properties. The results of the absolute humidity measurements correlate with those obtained from relative humidity assessments, affirming that the type and quantity of Tween utilized during the synthesis of oleogels significantly influence their moisture sorption properties.

### 3.3. Imaging of Oleogels via Optical Microscope

**Figure 5:**
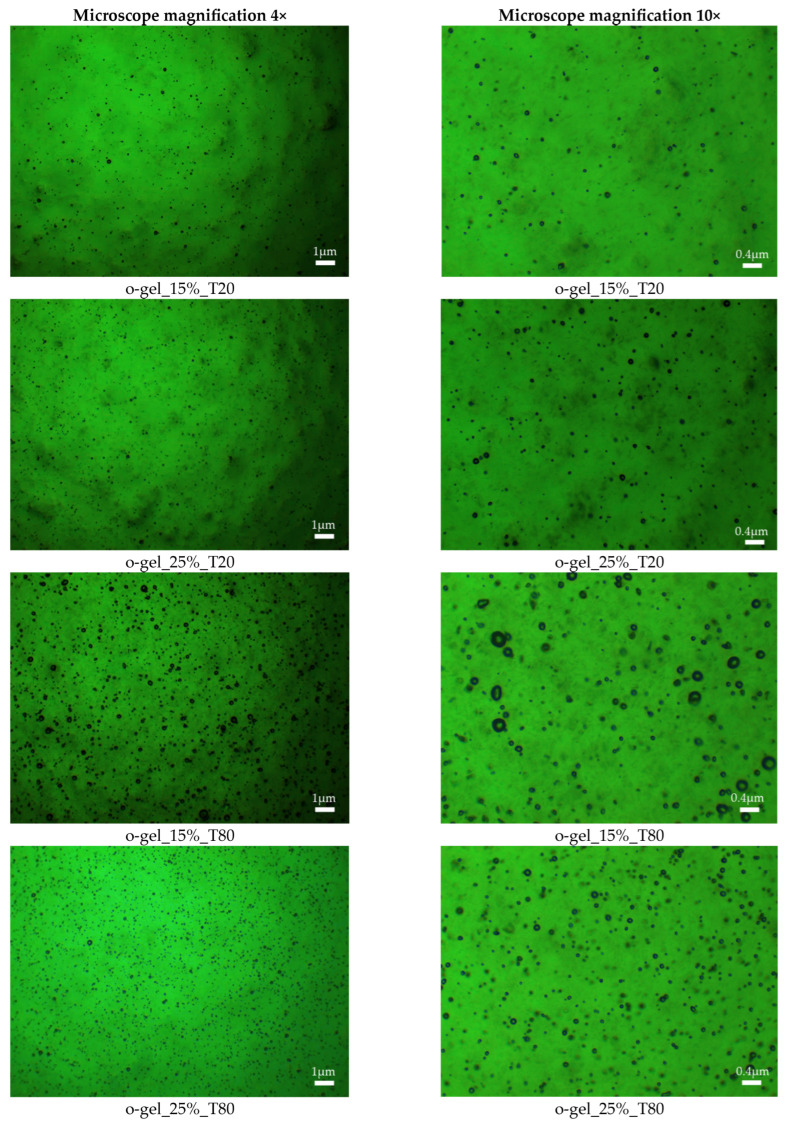
Optical microscope images of the obtained oleogel compositions in two magnifications, i.e., 4× and 10×.

Photographs of oleogels obtained using an optical microscope are shown below in Figure 5.

The optical microscope allowed for visualizing the morphology of the obtained materials. This analysis complemented the results obtained from surface wettability measurements and FT-IR spectroscopic analysis. The results indicate the influence of the type and amount of emulsifier on the morphology of the obtained oleogels, which was also observed in previous studies.

### 3.4. FT-IR ATR Spectroscopy

**Figure 6:**
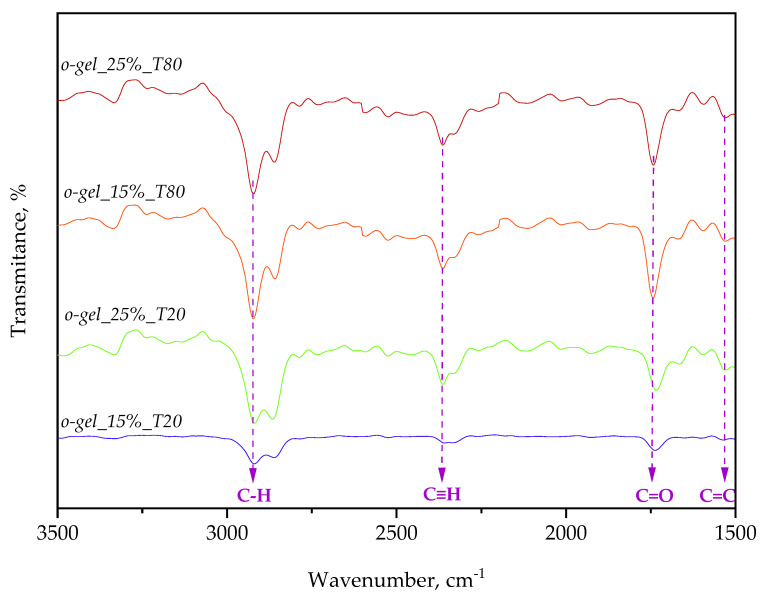
FT-IR ATR spectra of the obtained oleogels.

In Figure 6, FT-IR spectra of the oleogel samples are compiled; whereas in Table 4, the absorption bands visible on FT-IR spectra along with the vibration type and the corresponding functional groups are indicated.

FT-IR spectroscopy enabled the precise identification of the characteristic functional groups present in the investigated oleogel samples. Furthermore, the performed analysis allowed verification of the relationship between the content of emulsifying agents and the intensity of spectral bands.

### 3.5. Rheological Characteristics of Oleogels

**Figure 7:**
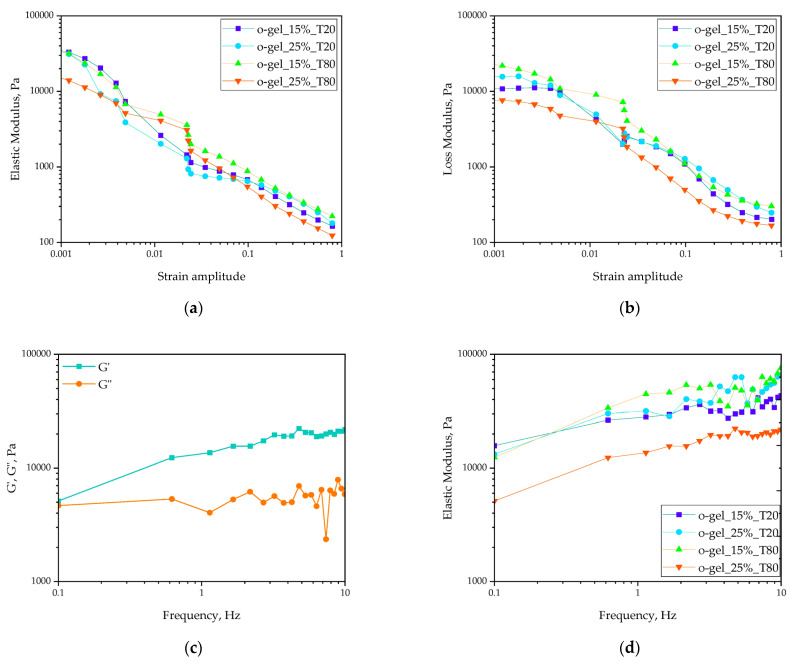
(**a**) Evolution of the elastic moduli with the applied strain amplitude. (**b**) Evolution of the loss moduli with the applied strain amplitude. (**c**) Evolution of the elastic and loss moduli in T80 25% oleogel with the frequency. (**d**) Evolution of the elastic moduli in all developed oleogels with the frequency.

In Figure 7a,b, the evolutions of the complex shear modulus’s real part (elastic modulus G′) and imaginary part (loss modulus G″) as a function of the applied strain amplitude for studied oleogels are presented. Figure 7c shows the evolution of the elastic and loss moduli in o-gel_25%_T80 with the frequency, while Figure 7d shows the evolution of the elastic moduli in all developed oleogels with the frequency.

## 4. Discussion

As part of the performed investigations, oleogels based on linseed and beeswax were obtained, stabilized using two non-ionic surfactants, i.e., Tween 20 and Tween 80. The parameter describing the ability of an emulsifier to interact with both the aqueous and oil phases is the HLB (hydrophilic–lipophilic balance) parameter [24,25,26]. In general, the higher the value of the HLB, the greater its hydrophilicity. The HLB value of Tween 20 is about 16.7, while that of Tween 80 is about 15. Hence, it can be concluded that Tween 20 demonstrates a slightly higher ability to reduce the interfacial tension between the components of the oleogel, thereby contributing to the formation of a uniform and stable oleogel, as well as to the uniform dispersion of its components. Moreover, oleogel prepared using Tween 20 may show a lower viscosity compared to oleogel prepared using Tween 80, as the uniform dispersion of oleogel components may ultimately lead to less friction between the particles, resulting in the lower flow resistance of such a system. Nonetheless, in order to verify the impact of the type of emulsifier used on the properties and stability of the oleogels obtained, two series of these materials were obtained using Tween 20 and Tween 80, i.e., emulsifying agents that differ in chemical structure.

**Figure 8:**
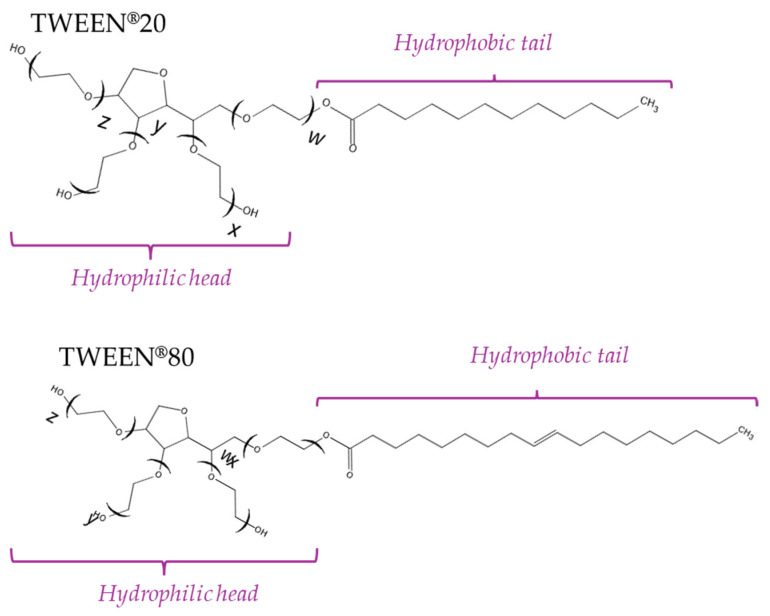
Chemical structures of both Tweens.

The results obtained from the wettability measurements showed a different effect of Tween 20 and Tween 80 content in the oleogel on the change in its hydrophilicity. As the content of Tween 20 in the oleogel increases, its hydrophilicity decreases. An inverse dependance was reported for Tween 80—an increase in its content in the test sample resulted in an increase in its hydrophilicity. These differences are most likely due to the difference in the chemical structure of Tweens—a scheme showing the chemical structures of both applied emulsifying agents indicating the difference in the size of their hydrophobic domains is presented below in Figure 8.

The key difference between the chemical structure of Tween 20 and Tween 80 is the number of ethylene oxide units in their hydrophilic domain. Tween 20 contains 20 ethylene oxide units, while Tween 80 contains 80. Ethylene oxide units are hydrophilic in nature; as a result, the more of them in a given structure, the greater that structure’s ability to interact with water, and therefore the more hydrophilic. In the case of Tween 80, which has four times more ethylene oxide units than Tween 20, its hydrophilic domain is richer in these units, making it more likely to interact with water. As a result, the higher the content of Tween 80 in an oleogel, the greater its hydrophilicity (lower wetting angle). Hence, it may be clearly stated that the type of Tween used during the synthesis of oleogels has a significant impact on their properties which was also reported in some other works [27,28].

Surface energy refers to the energy required to enlarge the surface area between two phases of a material. A lower surface energy means that the surface is more stable, meaning that it has less tendency to change its structure or separate into two phases. In other words, lower surface energy means that the surface has a greater ability to maintain its structure [28,29,30,31]. An analysis of surface energy suggests that a higher Tween content may have the effect of reducing or increasing the surface stability of oleogels. With an increase in the content of Tween 20 in the test sample, a decrease in surface energy and thus an increase in material stability is observed, while the opposite relationship is observed for Tween 80. The hydrophilic groups of Tween 20 interact with the wetting polar liquid, resulting in the emulsifier orienting the hydrophilic part towards this liquid and the hydrophobic part towards the hydrophobic components of the oleogel. This emulsifier layer can contribute to the better stabilization of the oleogel by reducing the interfacial tension between the oleogel and the wetting polar liquid. Lowering the interfacial tension between the oleogel and the wetting liquid acts as a barrier that hinders the entry of water into the oleogel, which maintains the stability of the system.

Relative humidity is a significant indicator characterizing the ability of the investigated materials to absorb water from the environment, thus representing their sorption properties. In the context of oleogels, these properties are crucial from the point of view of their stability and functionality, especially considering medical applications, such as delivering active substances. For instance, the high water absorption ability of oleogels from the surroundings can influence changes in their consistency over time, which in turn may result in the chaotic release of the active substance from oleogels.

Analyzing the obtained relative humidity values determined for the tested oleogels, one can find significant differences between individual samples, indicating the influence of both the type and the amount of Tween used in oleogel synthesis on their sorption properties. The initial higher relative humidity values for o-gel_25%_T80 and o-gel_15%_T80 samples compared to o-gel_25%_T20 and o-gel_15%_T20 samples suggest that Tween 80 tends to reduce the sorption capacity of oleogels.

Hence, the type of Tween used and its amount may be a key factor influencing the sorption properties of oleogels. This, in turn, relates to differences in the chemical structure between Tween 20 and Tween 80 which have been previously described in the discussion over the results of wettability measurements. Tween 80 has a longer hydrophobic fragment in its structure compared to Tween 20. As a result, the proportion of the hydrophilic part (and thus the proportion of hydrophilic functional groups) relative to the hydrophobic part in the structure of Tween 80 is smaller than in the structure of Tween 80, hence its lower sorption properties manifested by the lower absorption of water from the environment.

Thus, oleogels obtained with Tween 20 show less absorption of water from the surroundings than oleogels obtained with Tween 80, consequently demonstrating greater stability over time and greater functionality.

Spectral analysis of the oleogels demonstrated the presence of characteristic bands in the FT-IR spectra corresponding to functional groups present in the compounds comprising the oleogels, i.e., linseed oil, beeswax, and Tween. For example, the absorption band at 3333 cm^−1^ arises from stretching vibrations of the -OH bond, which is present in the structure of both Tween and the components of linseed oil and beeswax (e.g., fatty acids). Next, absorption bands visible at 3152 cm^−1^, 2919 cm^−1^, and 2857 cm^−1^ are characteristic for stretching vibrations of the -CH bond present in all organic compounds comprising the reagents used during oleogel synthesis. Furthermore, the band at 2361 cm^−1^ originates from the -C≡C- bond present in the structure of unsaturated fatty acids found in linseed oil. Additionally, the band at 1739 cm^−1^ arises from the C=O group present in the ester bond within the structure of beeswax. Moreover, the aforementioned chemical bonds (-CH, C=O, -OH) are also present in the structure of the emulsifying agents Tween 20 and Tween 80.

Importantly, it can be observed that with an increase in the content of the emulsifying agents in the investigated oleogel samples, the intensity of bands corresponding to individual functional groups also increases. Thus, spectral analysis confirmed the presence of chemical compounds in the analyzed oleogels, which are part of the reagents used during their synthesis, including linseed oil, beeswax, Tween 20, and Tween 80, with the intensity of the observed bands increasing with the content of the emulsifying agent in the oleogel.

The microscopic analysis of the obtained oleogels revealed that the addition of the emulsifier, specifically its chemical structure and quantity relative to the other components of the system, significantly influences the structure of the resulting materials. The micrographs suggest that a higher weight percentage of emulsifier with a longer hydrophobic tail leads to a denser structure of the material. It is important to emphasize that this observation is closely related to the stability of the materials over time and their subsequent properties. However, the characterization of the materials from this perspective will be the subject of a subsequent publication.

Based on the performed rheological experiments, it can be stated that the observed G′ and G″ curves present very similar values and courses regardless of the type of added surfactant and its concentration. However, it can be noticed that in the case of the highest tested concentration (25%) of Tween T80, the initial measured elasticity and loss moduli are significantly lower than in the case of the other samples. The obtained result suggests that the strength of developed oleogels decreased with the increasing surfactant concentration.

The amplitude sweep test was used to determine the linear viscoelastic range, which is the period during which the material subjected to the shear strain exhibits natural behavior without damage within the structure. As can be seen, any linear regimes on the elasticity G′ experimental curves exist. It means that the developed oleogels do not retain the original structure even for very low strain amplitudes, and the studied oleogels behave as liquid-like viscoplastic materials. As the amplitude increases, the elastic modulus decreases and finally becomes smaller than the loss modulus, which indicates that the materials already have a liquid-like behavior. Based on the methodology developed by [32] in the foam rheology studies, the materials’ yield stress as the intersection of the elastic and loss moduli curves can be determined. Most of the tested materials reach the yield stress with a strain amplitude of ca. 4 × 10^−3^ strain. Only in the case of o-gel_25%_T80 was the yield determined for the much higher value of strain amplitude (ca. equal to 0.2, see Supplementary Materials: Figures S1–S4).

The selected frequency sweep plot for o-gel_25%_T80 proves that the gel networks are characterized by physical cross-links (i.e., G′ > G″ independent of the frequency) for the studied oleogels (Figure 7c). Moreover, based on the results presented in Figure 7d, it can be reported that the dependence of elasticity moduli on frequency for all studied oleogels proves the fact that increasing concentration and that the change in surfactant from Tween 20 to Tween 80 decreases the strength of the materials.

The results obtained during the conducted research indicate the potential use of these materials in the fields of cosmetology and medicine. The ability to easily modify the structure and properties of the material depending on the emulsifier used provides great prospects for further studies, especially regarding the incorporation of medicinal substances into such systems, which will undoubtedly be the subject of our future research.

## 5. Conclusions

The conducted research revealed that the content of Tween 20 and Tween 80 significantly influenced the physicochemical properties of oleogels. A higher content of Tween 20 resulted in the decreased hydrophilicity of the oleogel, as evidenced by the reduction in the wetting angle (e.g., sample o-gel_25%_T20), whereas a higher content of Tween 80 exhibited the opposite effect, increasing the hydrophilicity of the samples, observed in o-gel_25%_T80. Surface energy analysis indicated that a higher content of Tween 20 reduced the surface energy of the oleogel, suggesting improved stability, as confirmed by the lower values of surface free energy in samples containing Tween 20. Additionally, changes in relative humidity values suggested a potential reduction in water absorption by oleogels containing Tween 80. FT-IR analysis confirmed the presence of characteristic absorption bands corresponding to functional groups present in the components of oleogels. Microscopic imaging of the material’s structure showed that a higher content of emulsifier with longer hydrophobic tails led to a denser structure of the oleogel. Rheological studies demonstrated that increasing the concentration of Tween 80 led to a decrease in the strength of oleogels, indicated by lower initial values of loss moduli and elasticity. Moreover, amplitude sweep experiments revealed that the obtained oleogels exhibited liquid-like behavior even at low strain amplitudes, without linear regimes on the elasticity curves. Conversely, frequency sweep analysis suggested that the strength of oleogels decreased with increasing surfactant content, shifting towards more liquid-like behavior, particularly noticeable when transitioning from Tween 20 to Tween 80. It is worth noting that oleogels maintained a solid consistency in the absence of stress; however, they exhibited the ability to spread over the surface after applying stress above approximately 25 Pa. These findings suggest potential applications of oleogels in cosmetics and medicine, particularly in modifying the structure and properties of the material depending on the type of emulsifier used.

Nonetheless, the presented studies are of a preliminary nature. Hence, more advanced investigations are needed to verify the influence of components’ proportions on the physicochemical and biological properties of the final material, optimize synthesis conditions, modify oleogels for specific applications, and study their stability under various environmental conditions and its impact on active substance transport and release.

## Figures and Tables

**Table 1:** Compositions of obtained oleogels.

Sample	Sample Name	Beeswax, g	Linseed Oil, g	Tween 20, *wt*/*wt*%	Tween 80, *wt*/*wt*%
1	o-gel_15%_T20	8	40	15	-
2	o-gel_25%_T20	8	40	25	-
3	o-gel_15%_T80	8	40	-	15
4	o-gel_25%_T80	8	40	-	25

**Table 2:** Representative photographs of water drop contact with the material layer at the first second and after complete spreading of the droplet on the material, i.e., after 10 seconds of measurement duration.

Name of Sample	First Contact of the Drop with the Material	Complete Spillage of the Drop on the Material after 10 s
o-gel_15%_T20	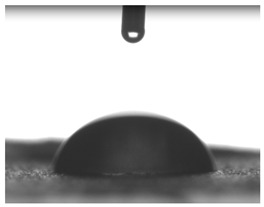	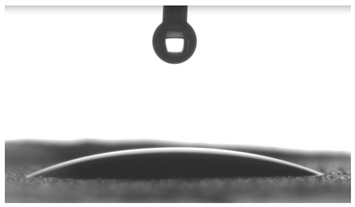
o-gel_25%_T20	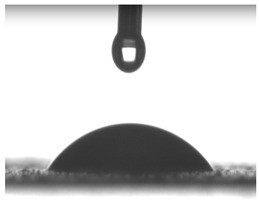	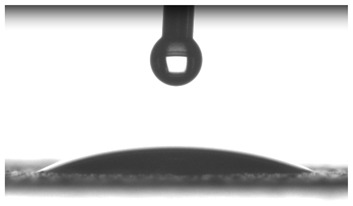
o-gel_15%_T80	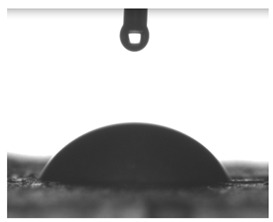	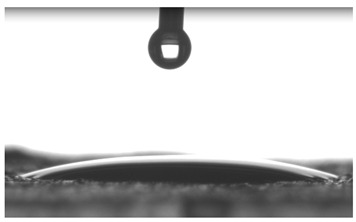
o-gel_25%_T80	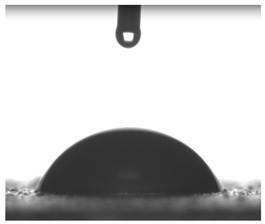	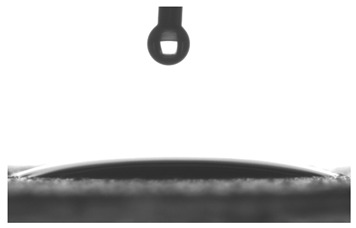

**Table 3:** Comparison of contact angles for distilled water and diiodomethane, along with the calculated surface energies based on their values.

Sample Name	Contact Angle, ° Average Value		Surface Free Energy		
	Distilled Water	Diiodomethane	Polar, mJ/m^2^	Dispersive, mJ/m^2^	Total Free Energy, mJ/m^2^
o-gel_15%_T20	17.0 +/− 0.089	10.7 +/− 0.667	34.41	39.45	73.86
o-gel_25%_T20	21.3 +/− 0.778	12.0 +/− 2.222	32.85	39.62	72.48
o-gel_15%_T80	22.1 +/− 2.867	11.6 +/− 0.756	32.43	39.81	72.24
o-gel_25%_T80	14.0 +/− 0.978	6.7 +/− 2.044	35.03	39.81	74.84

**Table 4:** Wavenumbers of observed absorption bands along with the vibration type and the corresponding functional groups.

Wavenumber, cm^−1^	Vibration Type	Functional Group
3333	stretching	-OH
3152	stretching	C-H
2919	stretching	C-H
2857	stretching	C-H
2361	stretching	-C≡C-
1739	stretching	C=O
1526	stretching	-C=C-

## Data Availability

The data presented in this study are available on request from the corresponding authors.

## Supplementary Materials

The following supporting information can be downloaded at: https://www.mdpi.com/article/10.3390/pharmaceutics16050600/s1, Figure S1. Transition point between liquid and solid states for o-gel_15%_T20. Figure S2. Transition point between liquid and solid states for o-gel_25%_T20. Figure S3. Transition point between liquid and solid states for o-gel_15%_T80. Figure S4. Transition point between liquid and solid states for o-gel_25%_T80.
