# Supplementary material for: Linseed Oil-Based Oleogel Vehicles for Hydrophobic Drug Delivery—Physicochemical and Applicative Properties
Source: Pharmaceutics. 2024 Apr 29;16(5):600. doi: 10.3390/pharmaceutics16050600 (PMC11125216; doi:10.3390/pharmaceutics16050600)
Supplement: Supplementary file 1 [file pharmaceutics-16-00600-s001.zip › pharmaceutics-2957761-supplementary.pdf]

pharmaceutics

Article

Linseed Oil-Based Oleogel Vehicles for Hydrophobic Drug Delivery – Physicochemical and Applicative Properties

Sonia Kudłacik-Kramarczyk [1,*], Anna Drabczyk [2,*], Alicja Przybyłowicz [1,3] and Marcel Krzan [1]

1 Jerzy Haber Institute of Catalysis and Surface Chemistry, Polish Academy of Sciences, 8 Niezapominajek St., 30-239 Krakow, Poland; marcel.krzan@ikifp.edu.pl (M.K.)
2 CBRTP SA - Research and Development Center of Technology for Industry, Ludwika Waryńskiego 3A St., 00-645 Warsaw, Poland
3 Faculty of Mechanical Engineering, Cracow University of Technology, Cracow University of Technology, 37 Jana Pawła II Av., 31-864 Krakow, Poland; alicja.przybylowicz@student.pk.edu.pl (A.P.)
* Correspondence: sonia.kudlacik-kramarczyk@ikifp.edu.pl (S.K.-K.), anna.drabczyk@cbrtp.pl (A.D.)

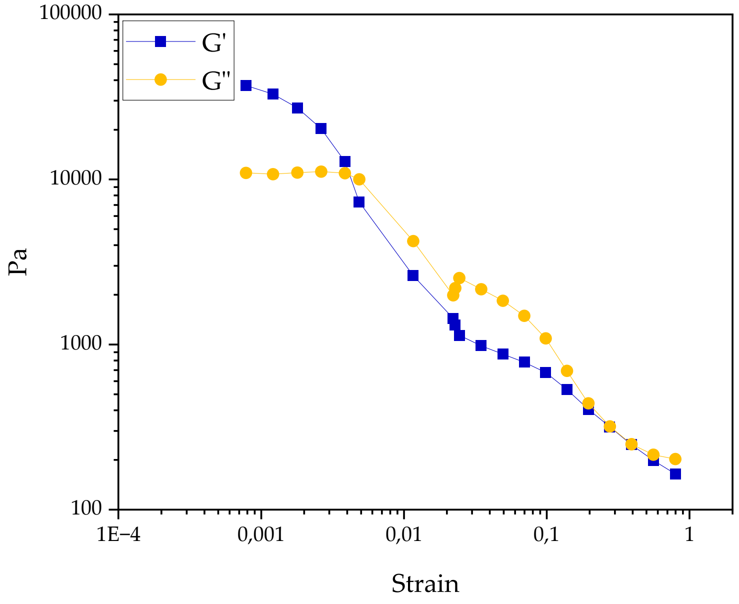

Figure S1. Transition point between liquid and solid states for o-gel_15%_T20.

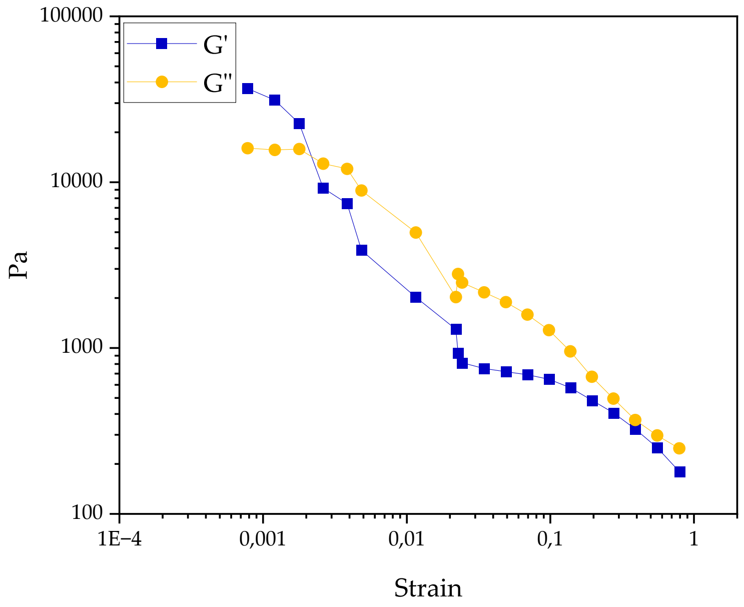

Figure S2. Transition point between liquid and solid states for o-gel_25%_T20.

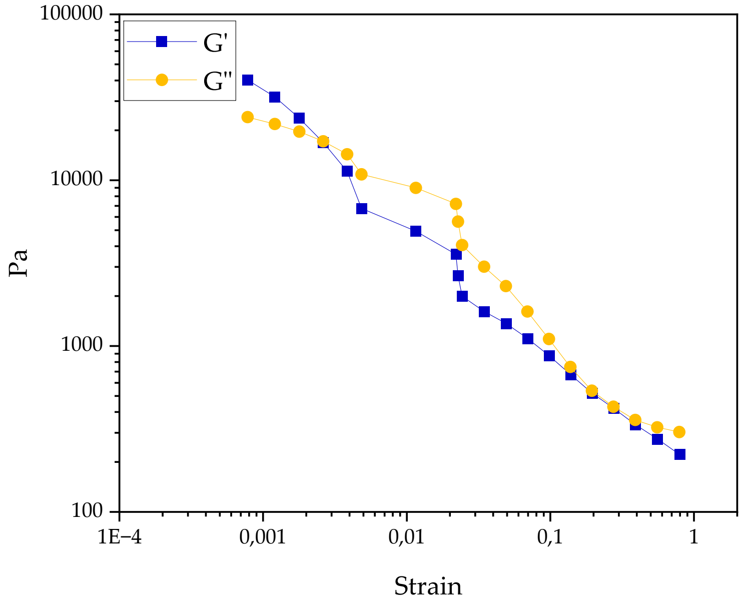

Figure S3. Transition point between liquid and solid states for o-gel_15%_T80.

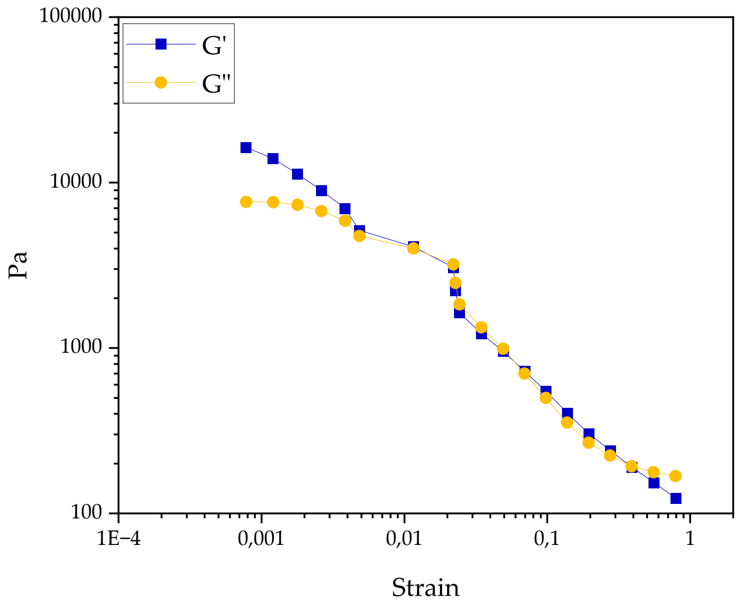

Figure S4. Transition point between liquid and solid states for o-gel_25%_T80.
